# Wheat Apoplast-Localized Lipid Transfer Protein *TaLTP3* Enhances Defense Responses Against *Puccinia triticina*

**DOI:** 10.3389/fpls.2021.771806

**Published:** 2021-11-25

**Authors:** Jiaojie Zhao, Weishuai Bi, Shuqing Zhao, Jun Su, Mengyu Li, Lisong Ma, Xiumei Yu, Xiaodong Wang

**Affiliations:** ^1^State Key Laboratory of North China Crop Improvement and Regulation, College of Plant Protection, Hebei Agricultural University, Baoding, China; ^2^College of Horticulture, Hebei Agricultural University, Baoding, China; ^3^College of Life Sciences, Hebei Agricultural University, Baoding, China

**Keywords:** wheat, pathogenesis-related protein, lipid transfer protein, apoplastic, resistance

## Abstract

Plant apoplast serves as the frontier battlefield of plant defense in response to different types of pathogens. Many pathogenesis-related (PR) proteins are accumulated in apoplastic space during the onset of plant–pathogen interaction, where they act to suppress pathogen infection. In this study, we found the expression of *Triticum aestivum* lipid transfer protein 3 (*TaLTP3*) gene was unregulated during incompatible interaction mediated by leaf rust resistance genes *Lr39/41* at the early infection stage. Stable transgenic wheat lines overexpressing *TaLTP3* exhibited enhanced resistance to leaf rust pathogen *Puccinia triticina*. Transcriptome analysis revealed that overexpression of *TaLTP3* specifically activated the transcription of pathogenesis-related protein 1a (TaPR1a) and multiple plant hormone pathways, including salicylic acid (SA), jasmonic acid (JA), and auxin, in response to the infection of the model bacterial pathogen *Pseudomonas syringae* pv. *tomato* DC3000. Further investigation indicated that *TaLTP3* physically associated with wheat *TaPR1a* protein in the apoplast. Transgenic wheat lines overexpressing *TaLTP3* and *TaPR1a* showed higher accumulations of reactive oxygen species (ROS) during plant defense responses. All these findings suggested that *TaLTP3* is involved in wheat resistance against leaf rust pathogen infection and forming a *TaLTP3-TaPR1a* complex in apoplast against this pathogen, which provides new insights into the functional roles of PR proteins.

## Introduction

Plants utilize complex defense mechanisms to fight against pathogen infections. Apoplastic space represents an essential site for pathogen infection and the frontier battlefield of plant defense response, where either plant-secreted proteins, such as proteases and pathogenesis-related (PR) proteins, directly interact with pathogens or cell surface-localized pattern recognition receptors (PRRs) perceive pathogen-associated molecular patterns (PAMPs) to initiate PAMP-triggered immunity (PTI) (Jashni et al., 2015; Qi et al., 2017). PR protein-encoding genes are highly induced by pathogen infections, leading to the enrichment of PR proteins in the apoplast that participate in various plant defense responses (Van Loon et al., 2006; Wang et al., 2018). It has been documented that induction of *PR* genes expression is considered as the indicator of the activation of phytohormone signaling pathways such as salicylic acid (SA) and jasmonic acid (JA) (Van Loon et al., 2006). PR proteins and peptides have also been utilized as promising tools for engineering plants with multiple stress tolerance (Ali et al., 2018).

Among all the designated PR proteins, *PR1* can be induced by various stresses and considered as a hallmark of plant defense responses with multiple functions (Breen et al., 2017). A quantitative peptidomics study revealed that the CAP-derived peptide 1 (CAPE1) peptide cleavage from C-terminus of *PR1* protein acts as a damage-associated molecular pattern (DAMP) triggering plant defense responses such as the burst of reactive oxygen species (ROS) and expression of other *PR* genes (Chen et al., 2014). More recently, a study showed that *TaPR1*-mediated host defense in wheat against septoria nodorum blotch (SNB) (caused by *Parastagonospora nodorum*) requires the cleavage of the C-terminal region, while toxin 3 (SnTox3) effector suppresses *TaPR1*-mediated plant defense by preventing the cleavage of the C-terminal region via direct interaction with overexpression of pathogenesis-related protein 1a (TaPR1a) (Sung et al., 2021). Interestingly, a recent investigation demonstrated that wheat *TaPR1* associates with thaumatin-like protein TaTLP1 [also known as pathogenesis-related protein 5 (TaPR5)] in the apoplastic space and exerts enhanced antifungal activities (Wang et al., 2020). Overexpression of wheat homolog of *PR1* gene (*TaPR1a*) in transgenic wheat line resulted in significantly enhanced resistance against both stripe rust and leaf rust fungi (Bi et al., 2020). Interestingly, they found that the model bacterial pathogen *Pseudomonas syringae* (*P. syringae*) pv. *tomato* DC3000 was sufficient to activate the transcriptional response of *TaPR1a*-mediated plant defense.

Lipid transfer proteins (LTP) belonging to PR14 family constitute a large protein family that exist in all the land plants and they are involved in various biological processes (Salminen et al., 2016). A growing body of evidence showed that LTPs are involved in plant defense against biotic stress. An *Arabidopsis* apoplastic LTP protein encoded by *defective in induced resistance 1* (*DIR1*) is proposed to interact with a lipid-derived molecule to trigger long-distance signaling (Maldonado et al., 2002). The expression of *Arabidopsis LTP3* is directly regulated by the transcription factor MYB96 (Guo et al., 2013), the latter which serves as a key transcriptional regulator bridging signal pathways of abscisic acid (ABA) and SA (Seo et al., 2009; Seo and Park, 2010). Barley lipid transfer protein 4 (*HvLTP4*), firstly designated as *PR14*, is highly induced by infection of powdery mildew (Molina and García-Olmedo, 1993). Several *Pichia pastoris*-expressed wheat *LTPs* showed direct antifungal activities against multiple pathogens (Sun et al., 2008). Wheat non-specific LTPs exhibit differential inhibition to wheat and non-wheat pathogens *in vitro* (Sun et al., 2008). Overexpression of wheat LTP gene *TaLTP5* results in increased resisance against *Cochliobous sativus* and *Fusarium graminearum* (Zhu et al., 2012). Wang et al. showed that overexpression of *Triticum aestivum* lipid transfer protein (*TaLTP3*) in *Arabidopsis* confers heat stress tolerance (Wang et al., 2014). More recently, heterologous overexpression of *Arabidopsis AtLTP4.4* in transgenic wheat results in enhanced resistance to *Fusarium* head blight and detoxification of mycotoxin deoxynivalenol (DON) (McLaughlin et al., 2020). Although wheat LTPs have been widely studied, the molecular mechanism underlying LTP-mediated defense response in apoplast remains unclear.

Our previous studies showed that *TaLTP3* protein was targeted by a wheat rust effector PNPi (Puccinia NPR1 interactor) (Bi et al., 2020). Therefore, we hypothesized that *TaLTP3* might play an important role in wheat resistance against rust infections. In this study, we generated transgenic wheat lines overexpressing *TaLTP3* to explore its fuctions during wheat defense responses and found transgenic lines exhibited enhanced resistance to leaf rust. Resistance mechanism of *TaLTP3* was further investigated by transcriptome sequencing. *TaLTP3* localized to the apoplast and interacted with *TaPR1a* suggesting that a *TaLTP3-TaPR1a* protein complex is involved in wheat resistance against leaf rust pathogen.

## Materials and Methods

### Gene Cloning, Generation of Wheat Transgenic Lines, and Pathogen Inoculation

The complete coding region of *TaLTP3* (GenBank accession AY226580.1) was amplified from complementary DNA (cDNA) synthesized from RNA extracted from the seedlings of wheat line “Chinese Spring.” The complete coding region of *TaLTP3* was constructed to binary T-DNA vector pLGY-02 (*Ubi:gene*, T-DNA). Transgenic wheat line overexpressing *TaLTP3* gene was generated in the genetic background of spring common wheat cultivar “JW1” (selected from a segregating population of the cross of spring common wheat cultivars “Fielder” and “NB1”) with technique support from Bangdi Ltd. Company, Shandong, China. Transgenic wheat lines overexpressing *TaPR1a* in the same genetic background were derived from a previous study (Bi et al., 2020).

Seedlings of the wheat isogenic line carrying leaf rust resistance gene *Lr39/41* in background of common wheat cultivar “Thatcher” were inoculated with *Puccinia triticina* (*Pt*), a virulent pathotype THTT following previously described procedure (Li et al., 2018). A high-resistant phenotype with the hypersensitive response (HR) was observed in *Lr39/41* isogenic line after inoculation with *Pt* pathotype THTT. RNA samples for quantitative PCR (qPCR) were collected from wheat leaves inoculated with leaf rust or water control at 0, 1, 2, 5, and 8 days postinoculation (dpi). Four independent biological replicates were included for each time point and treatment. Seedlings of transgenic wheat line *TaLTP3-OE* and wild-type plants were inoculated with *Pt* virulent pathotype FHJQ following a similar procedure. The wild-type plant “JW1” showed a moderate susceptible phenotype (rust sporulation with necrotic spots on wheat leaf) toward most of the collected *Pt* pathotypes including FHJQ and THTT. The disease symptoms were photographed at 14 dpi. An image analysis software ASSESS version 2.0 (American Phytopathological Society, St. Paul, Minisota, MN, United States) was employed to evaluate the percentage of *Pt* sporulation area for each inoculated leaf (Lamari, 2008; Bi et al., 2020). Two independent transgenic lines of *TaLTP3-OE* with 9–13 biological replicates were included for the *Pt* inoculation. The Dunnett’s test was conducted by using statistical analysis software SAS version 9.4.

Model bacterial pathogen *P. syringae* DC3000 was inoculated in second leaves of wheat seedlings according to the previously described method (Bi et al., 2020). *P. syringae* DC3000 was cultivated in low-salt Luria-Bertani (LS-LB) medium with 50 μg/mL rifampicin (Rif) antibiotics for 48 h (Jacob et al., 2017) and then diluted to optical density (OD) 600 = 0.6 in distilled water. Second leaves of transgenic wheat line *TaLTP3-OE* were infiltrated with bacterial suspensions by using a 1-ml needless syringe through the abaxial surface. Each transgenic line consisted of 6–8 independent biological replicates. Seedlings of wild-type plants and water-infiltration served as controls. The infiltration region was marked by using a marker pen. RNA samples for qPCR and RNA-sequencing (RNA-seq) assays were collected from the infiltration region at 6 h post-inoculation (hpi).

### Ribonucleic Acid-Sequencing

Ribonucleic acid samples were sent to Novogene Corporation Ltd. for KAPA library construction and transcriptome sequencing following the default procedure. The Illumina HiSeq 1000 System was employed for the 12-Gb sequencing for each RNA sample. Transcriptome was assembled with reference genome of *Triticum aestivum* (common wheat “Chinese Spring” TGACv1 version) (Appels et al., 2018) by using the HISAT2 FDR and StringTie software (Kim et al., 2019). Assembled transcripts that could not be found in the gene models of the reference genome were annotated as “novel” transcripts. Gene expressions were profiled by using the HTSeq version 0.9.1 software (Trapnell et al., 2010). The expected number of fragments per kilobase of transcript sequence per million base pairs (FPKM) for each gene was used to evaluate the expression of assembled genes. FPKM-based heatmaps were generated by the MeV software. Differentially expressed genes (DEGs) were filtered the expression levels of genes between different groups for an false discover rate (FDR)-adjusted *p*-value < 0.05 and |log2-fold change| > 1 by using the DESeq2 software (Love et al., 2014). The Gene Ontology (GO) annotations were assigned to each DEGs with the GO sequencing (Young et al., 2010). The KEGG (Kyoto Encyclopedia of Genes and Genomes) annotations were employed to profile the regulatory pathways enriched with DEGs (Kanehisa and Goto, 2000).

### Quantitative Real Time-PCR Assay

Ribonucleic acids were prepared by using the RNA Extraction Kit (QIAGEN, Hilden, Germany, United Kingdom) and the first-strand cDNA was synthesized by using the Reverse Transcription Kit (Takara, Dalian, China). Primers for qRT-PCR assay were designed (Supplementary Table 1) and a preliminary test was performed on the Roche LightCycler 96 qRT-PCR machine by using a series of 2-fold diluted cDNA templates (1:1, 1:2, 1:4, 1:8, and 1:32) to evaluate the amplification efficiency for each pair of primers. Melting curves were generated to ensure the specificity of the amplification. The wheat reference gene *TaActin* (GenBank accession AB181991) was used as the endogenous control (Paolacci et al., 2009). The transcript level was expressed relative to that of *TaActin* based on the 2^–ΔCt^ method as described in a previous study (Wu et al., 2019). Mean and SE were calculated by using the Microsoft Excel software. The two-sample *t*-test and the generalized linear model (GLM) ANOVA were performed by using the SAS software version 9.4.

### Subcellular Localization

Full-length open reading frame (ORF) of *TaLTP3* was cloned into transient expression vectors of pGWB5 (*35S:gene-GFP*, T-DNA) and pGWB454 (*35S:gene-RFP*, T-DNA), respectively. The recombinant vectors were transformed into *Agrobacterium* strain GV3101 and then used for infiltration of *Nicotiana benthamiana* (*N. benthamiana*). Corresponding empty vectors expressing only green fluorescent protein (GFP) and red fluorescent protein (RFP) served as controls. Green or red fluorescence was checked 48 hpi by using a Nikon Ti-2 microscope with a GFP or RFP filter, respectively. Epidermal peels of tobacco leaves were prepared from the infiltration region and soaked in 800 mM mannitol for 6 min to induce the plasmolysis (Ma et al., 2012).

### Yeast Two-Hybrid Assay

Primers that were used to amplify the full-length ORF of *TaLTP3* and five other plant defense-related *LTP* genes from cDNA synthesized from RNA isolated from the common wheat line “Chinese Spring” are listed in Supplementary Table 1. A neighbor-joint tree including all the LTP proteins in this study was generated by using the MEGA software version 7.0. The signal peptides of wheat LTPs were predicted by SignalP 5.0 server.^1^ The amplified DNA fragment lacking the sequence encoding the signal peptide was cloned into a Gateway yeast two-hybrid (Y2H) vector pLAW10 bait domain (BD). Recombined Y2H activation domain (AD) vectors carrying different regions of the *TaPR1a* gene were derived from a previous study (Bi et al., 2020). The coding regions of TaPR1a_(25–93aa)_ and TaPR1a_(25–128aa)_ were cloned into a Gateway Y2H vector pLAW11 (AD). Yeast transformation was performed by using lithium acetate and polyethylene glycol 3350 as previously described (Gietz and Schiestl, 2007). Cotransformed yeast strains were spotted on SD-Leu-Trp-His-Ade selective medium.

### Bimolecular Fluorescence Complementation Assay

Full-length ORF of *TaLTP3* with signal peptide was constructed to pDEST-^GW^VYNE (*35S:gene-YFP*^N^**, T-DNA). The recombinant construct of *TaPR1a*-pDEST-^GW^VYCE carrying complete coding region of *TaPR1a* was derived from our previous study (Bi et al., 2020). The fusion genes were coexpressed in tobacco leaves by *Agrobacterium* infiltration. Yellow fluorescence was checked 48 hpi by using a Nikon Ti-2 microscope with a yellow fluorescent protein (YFP) filter. Coexpression of *AvrLm1*-pDEST-^GW^VYNE and *BnMPK9*-pDEST-^GW^VYCE in the same vectors was used as a positive control (Ma et al., 2018). Signal peptide-fused empty vectors of *SP_(TaPR1*a)*_*-pDEST-^GW^VYNE and *SP_(TaPR1*a)*_*-pDEST-^GW^VYCE were derived from our previous study (Bi et al., 2020) and coexpressed with *TaPR1a*-pDEST-^GW^VYCE and *TaLTP3*-pDEST-^GW^VYNE, respectively, as negative controls.

### Co-immunoprecipitation Assay

Deoxyribonucleic acid fragment encoding the signal peptide-truncated *TaLTP3* protein was cloned into pGWB5 as *35S:TaLTP3_(30–122)_-GFP*. A specific reverse primer of *TaPR1a* with stop codon and sequence encoding cMYC tag was designed and used to amplify the DNA fragment encoding the signal peptide-truncated *TaPR1a* protein. The resulting DNA segment was constructed into pGWB5 as *35S:TaPR1a_(25–164)_-cMYC*. Recombinant pGWB5*:TaLTP3_(30–122)_-GFP* and pGWB5: *TaPR1a_(25–164)_-cMYC* were coexpressed in tobacco leaves by using *Agrobacterium*. Coexpression of *GFP* and *TaPR1a_(25–164)_-cMYC* served as a negative control. Total proteins were extracted from *Agrobacterium*-infiltrated tobacco leaves at 3 dpi and a portion of the total protein was reserved as an input sample. α-GFP IP samples were prepared by using GFP-trap beads (LABLEAD, Beijing, China). Input and IP samples were separated by sodium dodecyl sulphate-polyacrylamide gel electrophoresis (SDS-PAGE) and then transferred onto membranes made of polyvinylidene difluoride (PVDF). Immunoblots were performed by using α-GFP or α-cMYC antibody (Solarbio, Beijing, China).

### Reactive Oxygen Species Staining

To visualize the accumulation of ROS in wheat leaves infiltrated with *P. syringae* DC3000, two chemical reagents, nitroblue tetrazolium (NBT) and 3,3-diaminobenzidine (DAB), were applied to stain the superoxide anion (O_2_^–^) and hydrogen peroxide (H_2_O_2_), respectively. The staining protocols were modified from a previous study (Wang et al., 2007). Leaf regions infiltrated with *P. syringae* DC3000 were collected at 2, 4, and 8 hpi. For O_2_^–^ visualization, samples were soaked in 10 mM sodium azide (NaN_3_) and 10 mM potassium phosphate buffer (pH 7.8) with 0.1% NBT (w/v) for 24 h. For H_2_O_2_ staining, samples were incubated in hydrochloric acid (HCl)-acidified solution (pH 3.8) containing 1 mg/mL of DAB for 24 h. Thereafter, samples were decolored in boiling 95% ethanol for 10 min. The percentage of stained area in each leaf was determined by using the ASSESS software (Lamari, 2008). The whole experiment was repeated twice with two independent transgenic lines and each repeat consisted of 6–10 biological replicates. The Dunnett’s test was conducted by using the SAS software version 9.4.

## Results

### Stable Overexpression of *TaLTP3* in Transgenic Wheat Line Results in Enhanced Resistance to Leaf Rust Pathogen *Puccinia triticina*

**FIGURE 1:**
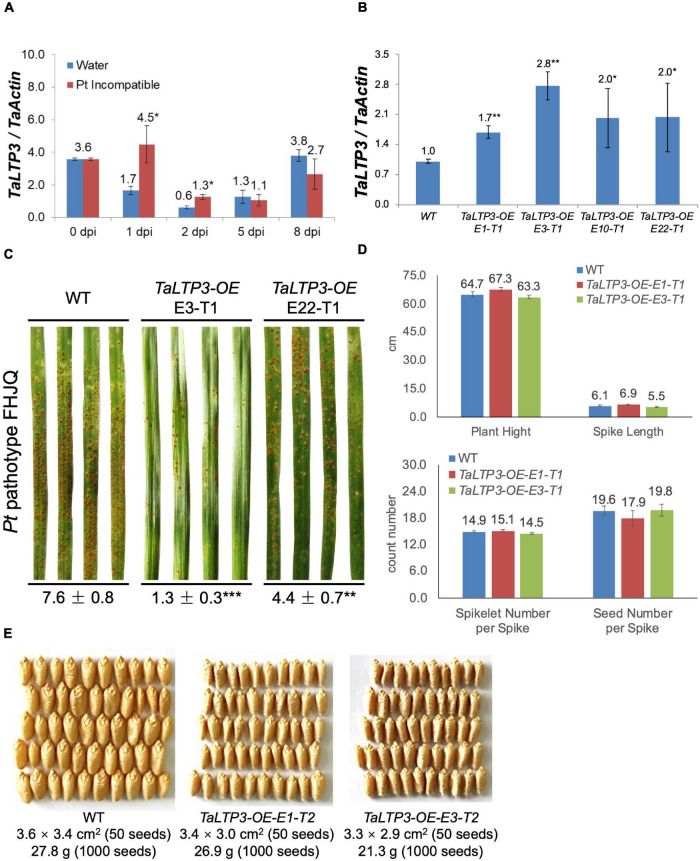
*TaLTP3* was involved in wheat resistance to leaf rust. **(A)** Expression levels of *TaLTP3* during *Lr39/41*-mediated wheat resistance toward leaf rust were evaluated by quantitative PCR (qPCR) assay. Leaf samples were collected at 0, 1, 2, 5, and 8 days postinoculation (dpi) with water (control) and avirulent *Pt* pathotype THTT. The transcript levels for all the genes were expressed as linearized fold-*TaActin* levels, which were calculated based on the formula 2^(Actin Ct^ ^–Target Ct)^. Data were expressed as mean ± SE from four biological replicates. The asterisk indicated a significant difference (*p* < 0.05) between the water control and *Pt*-inoculated samples by using the *t*-test. **(B)** The transcript level of *TaLTP3* in transgenic wheat line overexpressing *TaLTP3* and wild-type plants were evaluated by qPCR assay. **(C)** The susceptibility phenotypes of *TaLTP3-OE* and wild-type plants to the *Pt* pathotype FHJQ. The numbers below the images of the *Pt*-infected wheat leaves represent the average percentages of the rust sporulation area in each leaf. The asterisks (**p < 0.01, ***p < 0.0001) were annotated based on the comparison between the transgenic line and wild-type plants by the Dunnett’s test. **(D)** Agronomic traits of transgenic wheat line *TaLTP3-OE* and wild-type plants were investigated in greenhouse condition. **(E)** Seeds harvested from transgenic wheat line *TaLTP3-OE* and wild-type plants were compared in size and thousand grain weight.

To determine the role of *TaLTP3* in plant resistance, we first quantified the expression change of this gene in leaf rust resistance mediated by *Lr39/41* gene. Interestingly, *TaLTP3* was significantly upregulated at the wheat early-stage resistance toward leaf rust (Figure 1A). To further explore the function of *TaLTP3*, transgenic wheat lines overexpressing *TaLTP3* (*TaLTP3-OE*) were generated in the genetic background of commonspring wheat line “JW1.” The expression levels of *TaLTP3* in *TaLTP3-OE* reached 1.7- to 2.8-fold of those in the wild-type (Figure 1B). The third leaves of *TaLTP3-OE* and wild-type plants were inoculated with urediniospores of the *Pt* pathotype FHJQ and FHJQ exhibited high virulence on wild-type plants (Figure 1C). The sporulation of leaf rust pathogen was observed in both the *TaLTP3-OE* and wild-type plants. The percentage of *Pt* sporulation area for each inoculated leaf was evaluated by using the ASSESS software. Compared with wild-type plants, significantly enhanced resistance to *Pt* was observed in *TaLTP3-OE* as evidenced by decreased sporulation area (^**^p < 0.01, ^***^p < 0.0001, 9–30 biological replicates) (Figure 1C and Supplementary Table 2). We further investigated several agronomic traits of transgenic wheat line *TaLTP3-OE* including plant height, spike length, spikelet number per spike, and seed number per spike under greenhouse conditions. We did not observe any significant difference in these agronomic traits between *TaLTP3-OE* and wild-type plants (Figure 1D). However, when we checked the seed size and thousand grain weight, we found smaller and lighter seeds harvested from *TaLTP3-OE* than wild-type plants (Figure 1E), indicating overexpression of *TaLTP3* might have influence on wheat production.

### Overexpression of *TaLTP3* Positively Regulates Multiple Signaling Pathways Mediated by Plant Hormones of Auxin, Jasmonic Acid, and Salicylic Acid

**FIGURE 2:**
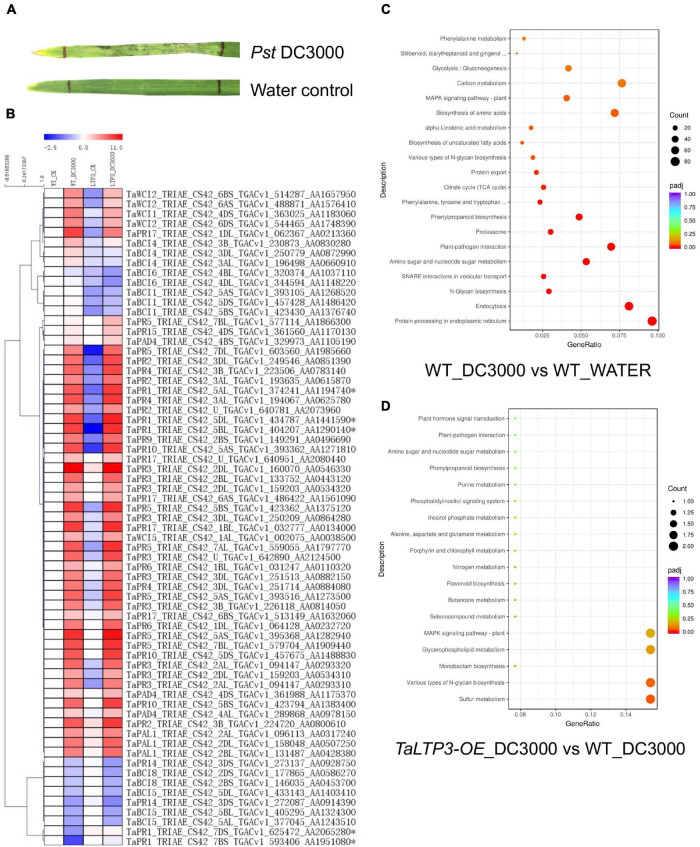
Transcriptome analysis revealed the regulatory network of *TaLTP3* during plant defense. **(A)** A typical cell death was observed in wheat leaf infiltrated with *P. syringae* DC3000 at 48 h postinfiltration. **(B)** Expression profile of *PR* genes upon *P. syringae* DC3000 infection in *TaLTP3-OE* transgenic wheat line. FPKM values of WT_DC3000, LTP_CK, and LTP_DC3000 were relative to that of WT_CK. Data were calculated as log2-fold change format by using the Microsoft Excel software. Heatmap was generated by using the MeV software. Genes with similar expression patterns were clustered. **(C,D)** The Gene Ontology (GO) annotations for upregulated differentially expressed genes (DEGs) in comparisons of “WT_DC3000 vs. WT_WATER”and “*TaLTP3-OE*_DC3000 vs. WT_DC3000.” DEGs were classified based on their GO annotations into three main categories including biological process, cellular component, and molecular function.

To reveal the mechanism of *TaLTP3* during plant resistance, we have used model bacterial pathogen *P. syringae* pv. *tomato* DC3000 to trigger the immune response in wheat leaves. Although *P. syringae* DC3000 is not a wheat pathogen under a natural environment, a typical cell death was observed in wheat leaf infiltrated with *P. syringae* DC3000 at 48 hpi (Figure 2A), indicating a successful induction of non-host resistance. We applied RNA-seq on samples collected from *P. syringae* DC3000-infiltrated third leaves of wheat transgenic line *TaLTP3-OE* and wild-type plants at 6 hpi. Samples from the water-infiltrated leaves served as a control. Each of the treatments included three biological replicates and, in total, 12 samples were subjected to a 12-Gb Illumina sequencing (Supplementary Table 3). The reference genome of *Triticum astivum* from the Ensembl Genomes (TGACv1 version) was used to assemble the transcriptome. A total of 122,130 transcripts were mapped to the genome sequence (Supplementary Table 4). Significant (*R*^2^ > 0.92) correlations of the overall gene expressions among biological replicates were detected (Supplementary Table 5). We used the FPKM value to predict the expression levels of transcripts. Raw reads from the RNA-seq were deposited at the National Center for Biotechnology Information (NCBI) BioProject PRJNA746113.

The expression levels of all 18 *PR* gene families were displayed in the heat map based on their FPKM values derived from the transcriptome data (Figure 2B). Most of the *PR* genes were highly induced by the infection of *P. syringae* DC3000, indicating an activation of a broad-range plant defense response by this model pathogen. Compared with the wild-type plants, *TaPR1a*, *TaPR5*, and *TaPR15* showed higher levels of expression in *TaLTP3-OE*. DEGs in comparison of “WT_DC3000 vs. WT_WATER” were identified (|log_2_-fold change| > 1 and *q*-value < 0.01) by using DESeq2. In the wild-type plants, we found 9,178 genes were significantly upregulated upon *P. syringae* DC3000 infection. The KEGG annotations of these *P*. *syringae* DC3000-sensitive genes indicated the activation of a broad-range plant defense responses including Ca^2+^-dependent ROS induction, mitogen-activated protein kinase (MAPK) signaling, and effector-triggered immunity (Supplementary Figure 1 and Supplementary Table 6). The GO annotations for these DEGs enriched in “plant–pathogen interaction” and “protein processing in endoplasmic reticulum” (Figure 2C).

From the comparison between “*TaLTP3-OE*_DC3000” and “WT_DC3000,” only 214 genes were significantly higher expressed (log_2_-fold change > 1 and *q*-value < 0.01) in *TaLTP3-OE* transgenic upon *P. syringae* DC3000 infection. The GO annotations of these upregulated DEGs enriched in “Various types of N-glycan biosynthesis” and “Glycerophospholipid metabolism” (Figure 2D). Among all these *TaLTP3*-responsive upregulated DEGs, *TaPR1a* was the only gene annotated into the KEGG pathway of “plant–pathogen interaction” (Table 1). Furthermore, we found 173 genes with significantly lower (log2-fold change < -1 and *q*-value < 0.01) expressions in *TaLTP3-OE* transgenic line upon *P. syringae* DC3000 infection. Several key regulators in the hormone pathway of auxin and JA, including *auxin response factor 9* (*ARF9*), *A-type Arabidopsis response regulator* (*A-ARR*), and *protein TIFY 6a* [JAZ (jasmonate ZIM-domain)], were downregulated in the KEGG pathway of “plant hormone signal transduction” (Table 1 and Supplementary Figure 2).

**TABLE 1:** DEGs of “LTP3_DC vs. WT_DC” annotated in the KEGG pathway of “plant–pathogen interaction” and “plant hormone signal transduction.”

**KEGG annotation**	**Gene ID**	**WT_DC FPKM**	**LTP3_DC FPKM**	**LTP3_DC vs WT_DC *p*-adjust value**	**Gene annotation**
PR-1	TRIAE_CS42_7BS_TGACv1_ 593406_AA1951080	17.26	63.11	4.34 × 10^–7^	Pathogenesis-related protein PRB1-2
ARF	novel.6352	1.21	0.03	1.01 × 10^–2^	Auxin response factor 9
A-ARR	TRIAE_CS42_2AL_TGACv1 _093101_AA0272020	19.57	11.16	2.72 × 10^–2^	Two-component response regulator ORR6
	TRIAE_CS42_2BL_TGACv1 _132162_AA0435700	3.14	0.71	6.55 × 10^–5^	Two-component response regulator ORR1
JAZ	TRIAE_CS42_5AL_TGACv1 _375081_AA1215750	2.74	0.66	1.13 × 10^–3^	Protein TIFY 6a

*LTP, lipid transfer protein; CK, mock inoculation with water; DC, P. syringae DC3000 infection; DEGs, differentially expressed genes.*

**FIGURE 3:**
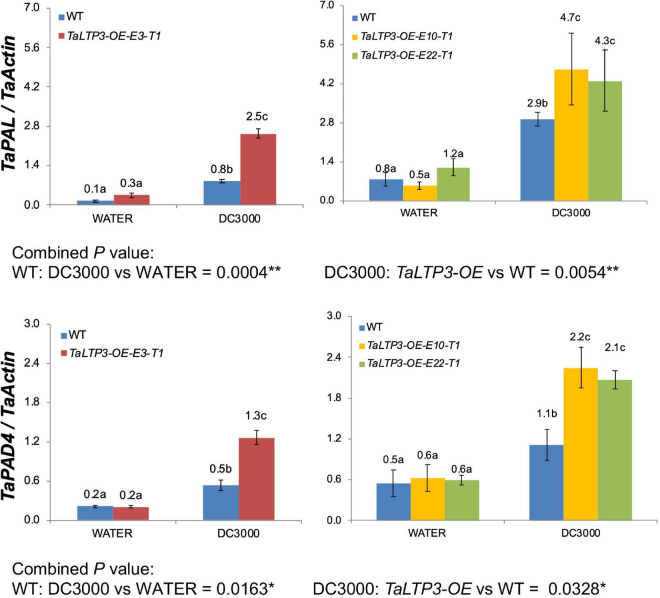
The transcriptional levels of *TaPAL* and *TaPAD4* were determined by qPCR in transgenic line *TaLTP3-OE* upon *P. syringae* DC3000 infection. The transcript levels for all the genes were expressed as linearized fold-*TaActin* levels, which were calculated based on the formula 2^(Actin Ct^ ^–Target Ct)^. Three independent transgenic lines of *TaLTP3-OE* were tested. Each experiment, consisting of 4–9 biological replicates, was considered as a block. Calculations of the mean and SE were performed by using the Microsoft Excel software. Generalized linear model (GLM) ANOVA (*p < 0.05, **p < 0.01) was conducted by using the SAS software version 9.4.

We further evaluated the expression levels of two SA synthesis genes, *TaPAL* and *TaPAD4*, during *P. syringae* DC3000 infection in *TaLTP3-OE* transgenic line by using qRT-PCR assay (Figure 3). qPCR showed that the expression levels of both the *TaPAL* and *TaPAD4* were elevated upon *P. syringae* DC3000 infection in *TaLTP3-OE* transgenic line, which was consistent with those in RNA-seq (Figure 2B), suggesting that our RNA-seq data are reliable.

### *TaLTP3* Interacts With Pathogenesis-Related Protein 1a in the Apoplastic Space

**FIGURE 4:**
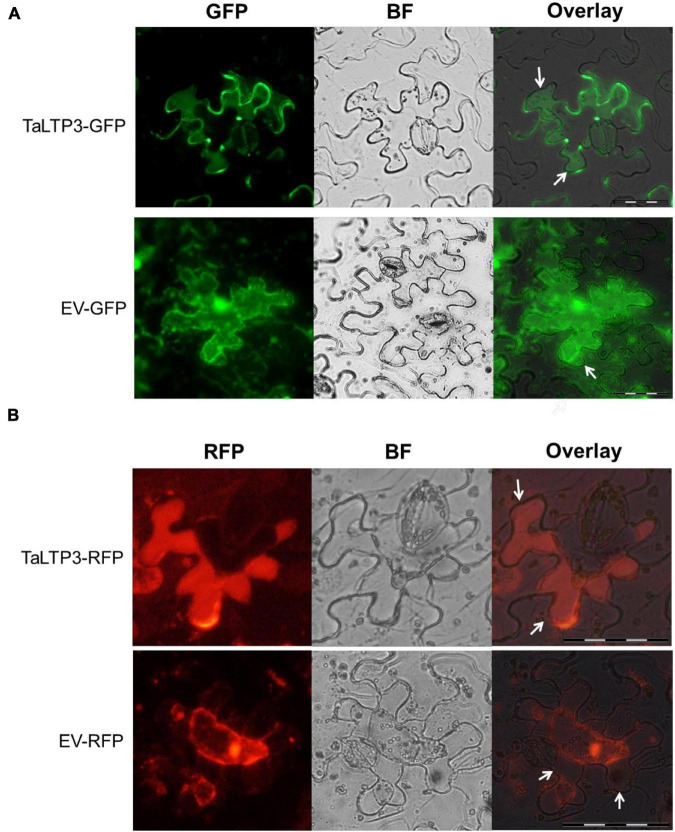
*TaLTP3* protein was localized in the apoplastic space. The *TaLTP3-GFP* **(A)** and *TaLTP3-RFP* **(B)** Recombinant proteins were transiently expressed in *N. benthamiana* leaves by agroinfiltration. GFP or RFP alone was used as a control. Leaf epidermal peels were plasmolyzed by incubation in 800 mM mannitol for 6 min. White arrows indicating the separation between the plant cell wall and plasma membrane during plasmolysis. Scale bar = 100 μm. BF, bright field; EV, empty vector; GFP, green fluorescent protein; RFP, red fluorescent protein.

As overexpression of *TaLTP3* affected *TaPR1a* expression during plant defense response (Figure 2B, Supplementary Figure 2, and Table 1) and both *TaLTP3* and *TaPR1a* are predicted as secreted proteins by signal P, we hypothesized that *TaLTP3* might function through physical interaction with *TaPR1a* in the apoplastic space. The apoplast localization of *TaPR1a* has been validated in our previous study (Bi et al., 2020). GFP- and RFP-tagged *TaLTP3* were generated, respectively. The recombinant constructs *TaLTP3-GFP* and *TaLTP3-RFP* were transientlyexpressed in *N. benthamiana* by agroinfiltration. Plasmolysis was induced by treatment with 800 mM mannitol. Pronounced green and red fluorescent signals for *TaLTP3-GFP* and *TaLTP3-RFP* were visualized in the apoplastic space, respectively (Figure 4), but not for the empty vector (EV-GFP and EV-RFP) controls (Figure 4), suggesting that *TaLTP3* localizes in the apoplastic space.

**FIGURE 5:**
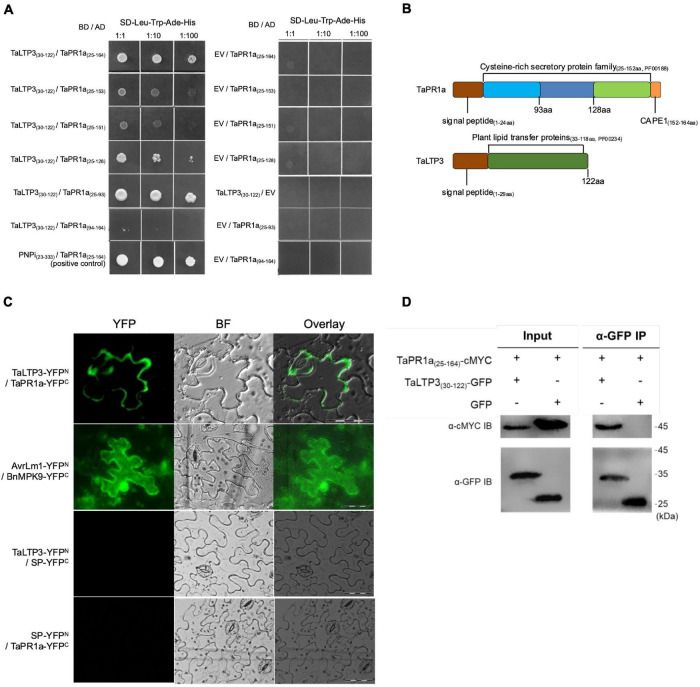
*TaLTP3* physically associated with *TaPR1a in vitro* and *in vivo*. **(A)** Interaction of *TaLTP3* and *TaPR1a* was detected by Y2H. Yeast transformants coexpressing different bait and prey recombinant constructs were tested on selective medium of SD-Leu-Trp-His-Ade, EV, and empty vector. **(B)** Signal peptide and conserved domain for *TaPR1a* and *TaLTP3* proteins were schematically presented. **(C)** Bimolecular fluorescence complementation (BiFC) assay showed that the interaction between TaPR1a and TaLTP3 occurred in the apoplastic space. YFP signals were observed in the tobacco leaves coexpressing *TaLTP3-YFP*^N^** and *TaPR1a-YFP*^C^**. Recombinant constructs of AvrLm1-YFP^N^ and BnMPK9-YFP^C^ were coexpressed as a positive control. Segment encoding signal peptide of *TaPR1a* was cloned into BiFC vectors as SP-YFP^N^ and SP-YFP^C^ as negative controls. Scale bar = 100 μm. Due to the fixed microscopy settings for visualizing yellow and green fluorescent signals, the yellow fluorescent signals were presented in green color. **(D)** Validation of the interaction between *TaPR1a* and *TaLTP3* by co-immunoprecipitation (Co-IP) assay. Total proteins from tobacco leaves transiently expressing *TaPR1a*_(25–164)_-cMYC and *TaLTP3*_(30–122)_-GFP were extracted and immunoprecipitated with an α-GFP antibody (α-GFP IP). Input and bound proteins were immunoblotted with α-GFP and α-cMYC antibodies, respectively. Original, uncropped blots for the Co-IP assay were displayed as Supplementary Figure 4. BF, bright field; EV, empty vector; YFP, yellow fluorescent protein; BiFC, bimolecular fluorescence complementation; Y2H, yeast-two hybrid.

To examine the interaction between *TaLTP3* and *TaPR1a*, a Y2H assay was performed by using a signal peptide-truncated version of these two proteins. Strong interaction was observed between *TaPR1a*_(25–164)_ and *TaLTP3*_(30–122)_ as yeast transformed with both the constructs was able to grow on the stringent synthetic dropout (SD) selective media lacking leucine, tryptophan, histidine, and adenine (SD-Leu-Trp-His-Ade) (Figure 5A). Because the C-terminal region of *TaPR1a* contains the conserved CAPE1 peptide in most PR1 proteins that might act as a DAMP in triggering plant defense response, we generated several truncated versions of *TaPR1a* protein, in which different C-terminal regions were deleted (Figure 5B). As shown in Figure 5A, an N-terminal region of TaPR1a ranging from 25 to 93 aa was sufficient to mediate the interaction with *TaLTP3*. However, we did not observe any interaction between TaPR1a (94–164 aa) and *TaLTP3*. As many wheat *LTP* genes, including *TaLTP2*, *TaLTP3F1*, *TaLTP4.3*, *TaLTP4.1*, and *TaPR14*, have been previously reported in wheat resistance to various pathogens, we also checked the interaction of *TaPR1a* with these LTP proteins and found that *TaPR1a* interacts with all these LTP proteins (Supplementary Figure 3). Collectively, these initial Y2H results indicated a conserved physical interaction between *TaPR1a* and LTP proteins.

To validate the *in-planta* interaction between *TaLTP3* and *TaPR1a*, a bimolecular fluorescence complementation (BiFC) assay was performed in *N. benthamiana*. Strong yellow fluorescence signals in the apoplast were observed in *N. benthamiana* leaves coexpressing *TaLTP3-YFP*^N^** and *TaPR1a-YFP*^C^** (Figure 5C), whereas fluorescence in cytoplasm and nucleus was found in positive control coexpressing *AvrLm1-YFP*^N^** and *BnMPK9-YFP*^C^** (Ma et al., 2018). No YFP signals were observed in the *N. benthamiana* leaves coexpressing the negative controls: *TaLTP3-YFP*^N^** and *SP_(TaPR1*a)*_-YFP*^C^** nor *SP_(TaPR1*a)*_-YFP*^N^** and *TaPR1a-YFP*^C^**.

To further confirm the interaction *in vivo*, a co-immunoprecipitation (co-IP) assay was performed following transient expression of *TaPR1a_(25–164)_-cMYC* and *TaLTP3_(30–122)_-GFP* constructs in *N. benthamiana*. GFP was used as a negative control. cMYC-tagged TaPR1a co-immunoprecipitated with GFP-tagged TaLTP3, but not with GFP (Figure 5D and Supplementary Figure 4), indicating that *TaLTP3* physically interacts with *TaPR1a*.

### Overexpression of Pathogenesis-Related Protein 1a and *TaLTP3* in Wheat Increases Accumulation of Reactive Oxygen Species Triggered by *P. syringae* DC3000

**FIGURE 6:**
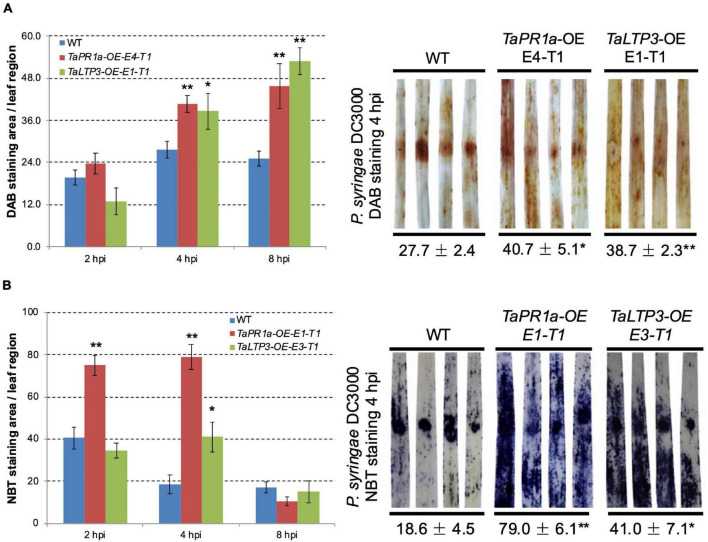
Transgenic wheat lines *TaPR1a-OE* and *TaLTP3-OE* showed higher accumulation of reactive oxygen species (ROS) upon infection of model bacterial pathogen *P. syringae* DC3000. **(A)** Samples from the infiltrated region were collected at 2, 4, and 8 hours postinoculation (hpi) and further stained with 3,3-diaminobenzidine (DAB). **(B)** Corresponding samples were stained with nitroblue tetrazolium (NBT) to visualize the accumulation of superoxide anion (O_2_^–^). The percentage of stained area in the infiltrated region was calculated By using the ASSESS software. Asterisk indicates the significance of the differences between the transgenic lines and wild-type plants established by using the Dunnett’s test (*p < 0.05, **p < 0.01, Supplementary Tables 7, 8).

To explore the coeffects of *TaLTP3* and *TaPR1a* during plant defense response, we evaluate the accumulation of H_2_O_2_ and O_2_^–^ in the transgenic wheat lines *TaLTP3-OE* and *TaPR1a-OE* during *P. syringae* DC3000 infection. Leaf samples were collected at 2, 4, and 8 h after *P. syringae* DC3000 inoculation and stained with DAB and NBT to visualize H_2_O_2_ and O_2_^–^, respectively. Percentage of the stained area in each infiltrated leaf was calculated by using the ASSESS software. Compared with the wild-type plants, we observed significantly higher (*p < 0.05, ^**^p < 0.01, 6–10 biological replicates) accumulation of H_2_O_2_ in the transgenic wheat lines *TaPR1a-OE* and *TaLTP3-OE* at 4 and 8 hpi (Figure 6A and Supplementary Table 7). For the NBT staining, the transgenic wheat line *TaPR1a-OE* showed a fast and higher accumulation of O_2_^–^ at 2 and 4 hpi, whereas the transgenic wheat line *TaLTP3-OE* accumulated more O_2_^–^ only at 4 hpi (Figure 6B and Supplementary Table 8). These results indicated that both the *TaLTP3* and *TaPR1a* might function through a ROS-dependent plant defense response.

## Discussion

Many designated PR proteins are secreted into the apoplastic space to function. Therefore, apoplast represents the initial battlefield between plant and phytopathogens interaction and apoplastic immunity plays an important role in plant defense responses. *TaLTP3*, a member of the *PR14* gene family, was upregulated during wheat resistance to leaf rust infections (Figure 1A). Our studies showed that both the *TaLTP3* and *TaPR1a* are secreted into the apoplastic space (Figure 4; Bi et al., 2020). PR1 protein functions in multiple ways during plant defense response including direct antimicrobe activity, triggering DAMP-triggered immunity, and targeting pathogen effectors (Chen et al., 2014; Gamir et al., 2017; Sung et al., 2021). LTP also has antifungal and antioxidant activities (McLaughlin et al., 2020). In *Arabidopsis*, LTP was reported as upstream of SA-mediated resistance to biotic and abiotic stresses (Maldonado et al., 2002; Guo et al., 2013). Previous studies showed that PR1 interacts with pathogenesis-related proteins from plants and effectors from pathogens (Breen et al., 2017), suggesting that PR1 has a broad-spectrum interactors in plant–pathogen interaction. Therefore, we speculated that PR1 might form different complexes with various plant defense-related proteins targeting phytopathogens. In this study, we identified and validated a direct interaction between *TaLTP3* and *TaPR1a* in the apoplastic space (Figure 5). Further study showed that this interaction was conserved among *TaPR1a* and other plant-defense-related LTP proteins (Supplementary Figure 3). The CAPE1 peptide in the C-terminal region of PR1 is conserved and acts as a DAMP-triggered plant defense-related transcriptional change (Chen et al., 2014). However, our studies showed the N-terminal region of *TaPR1a*, excluding the CAPE1 peptide, is required for the interaction between *TaLTP3* and *TaPR1a* (Figures 5A,B). We also found that the interaction of *TaLTP3* and *TaPR1a* occurs in the apoplast (Figure 5C). These observations implicated that TaLTP3 protein forms a complex with *TaPR1a* in the apoplast without occupying the CAPE1 peptide region of TaPR1a, suggesting that *TaLTP3* might stabilize *TaPR1a* via physical interaction to potentiate the CAPE1-triggered plant immune response. Further studies to determine our hypothesis are needed.

Our findings showed that transgenic lines *TaLTP3-OE* and *TaPR1a-OE* exhibited improved resistance to leaf rust (Figure 1C; Bi et al., 2020) and increased accumulation of ROS upon *P. syringae* DC3000 infection (Figure 6 and Supplementary Tables 7, 8). ROS has been widely reported to have a direct antimicrobial effect and can be involved in cell wall stiffening. Moreover, ROS acts as local and systemic signal molecules triggering plant defense response (Waszczak et al., 2018). Our results indicated that both the *TaPR1a* and *TaLTP3* may promote ROS accumulation during pathogen infection. A total of 330 *LTP* genes were identified from hexaploid genome of wheat and many of which were highly induced by abiotic stresses of drought and salinity (Fang et al., 2020). Close homologs in the *LTP* and *PR1* gene families might also have functional redundancy. Future studies focusing on dissecting the redundant functions between *LTP* and *PR1* genes are required.

In our previous study, we noticed that infection of model bacterial pathogen *P. syringae* DC3000 in wheat was sufficient to induce the expression of CAPE1-responsive genes *TaAdi3* and *TaPR7* (Bi et al., 2020). Therefore, we challenged the *TaLTP3-OE* line with model bacterial *P. syringae* for RNA-seq analysis. A specific upregulation of *TaPR1a* was detected in the transcriptome database by the KEGG annotation in *TaLTP3-OE* line compared to that in the WT plant (Table 1), suggesting that overexpression of *TaLTP3* can affact the expression of *TaPR1a* that may explain the earlier induction of *TaLTP3* than *TaPR1a* during wheat resistance to leaf rust mediated by the *Lr39/41* gene (Figure 1A; Li et al., 2018). Furthermore, we found suppressions of key regulator genes in auxin and JA pathways (*ARF9*, *A-ARR*, and *JAZ*) in *TaLTP3-OE* transgenic line (Table 1). Both the *ARF9* and *A-ARR* are negative regulators suppressing transcription of specific auxin-responsive genes (Leyser, 2006). JAZ proteins are repressors of JA signaling and inhibit the expression of JA-responsive genes by interaction with jasmonate-insensitive 1 (JIN1) transcription factor transcription factor (Chini et al., 2007). In addition, gene expressions of *TaPAL* and *TaPAD4* in upstream of SA pathway were highlyinduced in *TaLTP3-OE* transgenic line (Figures 2B and 3). Taken together, we speculated that *TaLTP3* acts as a positive regulator in the activation of crosstalk among SA, JA, and auxin. Although SA and JA are normally considered to control antagonistic pathways (Pieterse et al., 2009; Li et al., 2019), wheat plants seem to have a unique mechanism of hormone-mediated regulation of disease resistance. For instance, infection of spot blotch in resistance wheat genotype “Yangmai#6” elicited both the SA and JA signaling (Sahu et al., 2016). Spray treatments of both the SA and JA, but not ABA, on wheat leaves improved resistance to leaf rust (Kim et al., 2020).

## Conclusion

We validated a positive role of *TaLTP3* in plant defense response. Stable expression of *TaLTP3* in transgenic wheat line improved resistance to leaf rust infection. Overexpression of *TaLTP3* elevates the expression of *TaPR1a* and activates multiple plant defense-related hormone pathways including SA, JA, and auxin. A novel interaction between *TaLTP3* and *TaPR1a* in the apoplast was identified and confirmed. TaLTP3 might play multiple roles in plant defense response, such as functioning with antimicrobe activities, forming a *TaLTP3-TaPR1a* complex in apoplast against pathogen and activating defense signaling. The presented findings have greatly expanded our knowledge on the molecular mechanism of *PR* genes during wheat resistance to pathogen infections. The generated transgenic wheat lines with enhanced defense responses may also serve as valuable resources for the genetic improvement of wheat resistance.

## Data Availability Statement

The original contributions presented in the study are publicly available. This data can be found here: National Center for Biotechnology Information (NCBI) BioProject database under accession number PRJNA746113.

## Author Contributions

XW and LM designed the experiment. JZ and WB conducted most of the experiments. SZ, JS, and ML worked on the RT-qPCR validation and rust inoculation. XW drafted the manuscript with contributions from LM and XY. All the authors read and approved the final manuscript.

## Conflict of Interest

The authors declare that the research was conducted in the absence of any commercial or financial relationships that could be construed as a potential conflict of interest.

## Publisher’s Note

All claims expressed in this article are solely those of the authors and do not necessarily represent those of their affiliated organizations, or those of the publisher, the editors and the reviewers. Any product that may be evaluated in this article, or claim that may be made by its manufacturer, is not guaranteed or endorsed by the publisher.

## Footnotes

1
http://www.cbs.dtu.dk/services/SignalP/
