# Supplementary material for: Wheat Apoplast-Localized Lipid Transfer Protein TaLTP3 Enhances Defense Responses Against Puccinia triticina
Source: Front Plant Sci. 2021 Nov 25;12:771806. doi: 10.3389/fpls.2021.771806 (PMC8657149; doi:10.3389/fpls.2021.771806)
Supplement: Supplementary file 1 [file Data_Sheet_1.PDF]

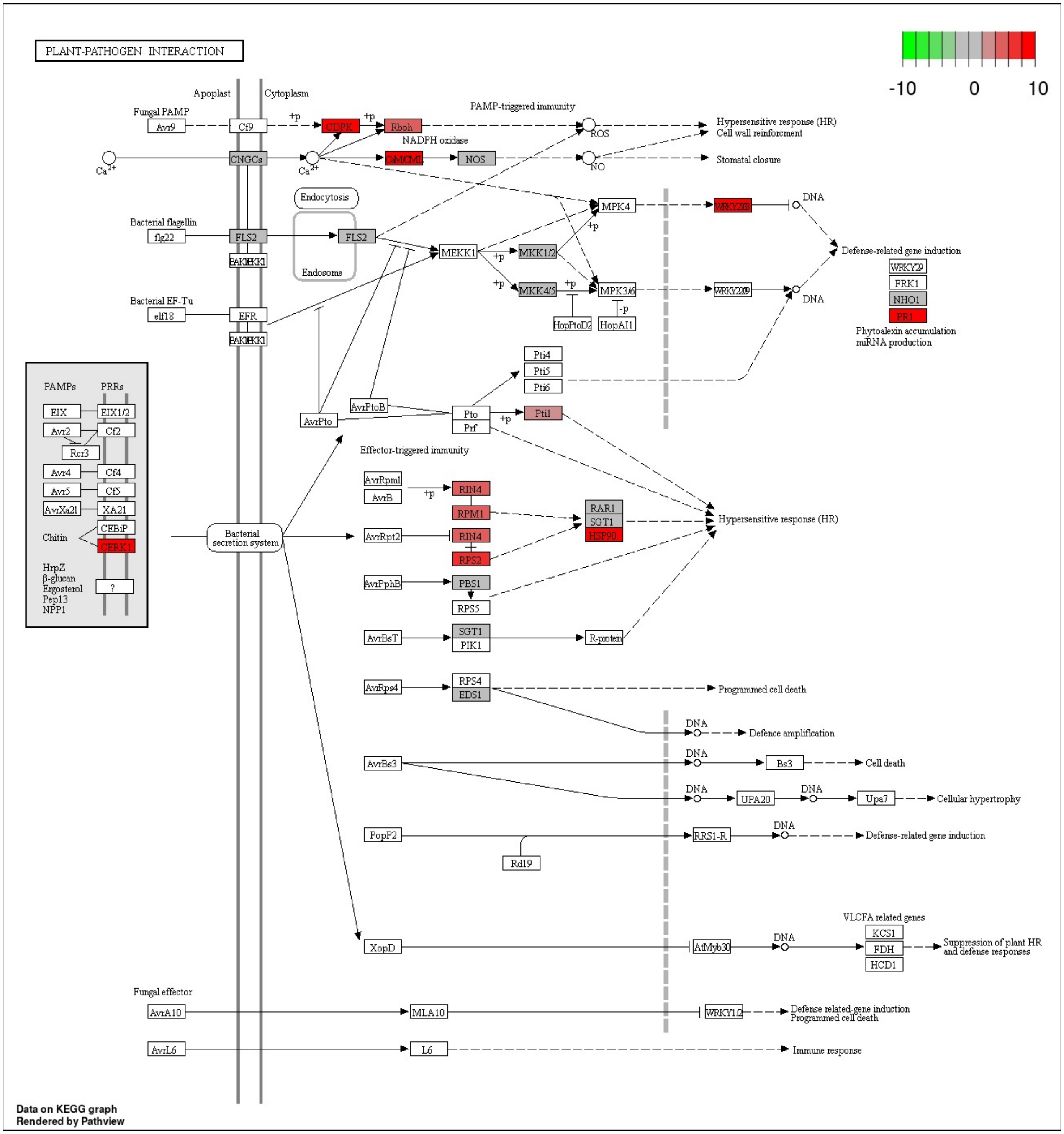

WT_DC3000 vs WT_WATER

SUPPLEMENTARY FIGURE S1 | The "plant-pathogen interaction" KEGG pathway annotations for the identified DEGs in comparison of "WT_DC3000 vs WT_WATER". Nodes of KEGG pathway enriched with significantly upregulated DEGs were framed in red color, and downregulated DEGs in green color. Detailed information in **Supplementary Table S6**.

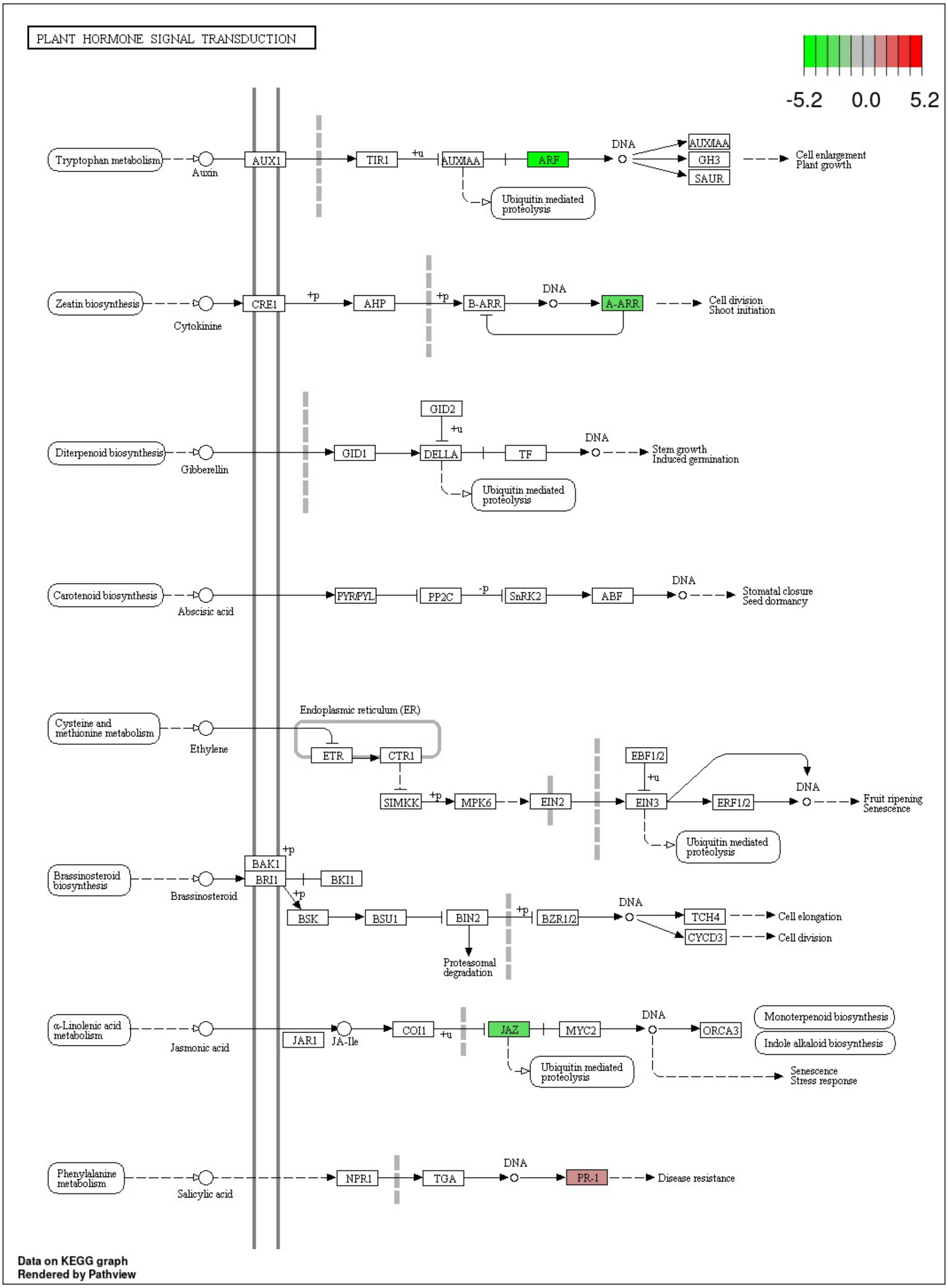

SUPPLEMENTARY FIGURE S2 | The “plant hormone signal transduction” KEGG pathway annotations for the identified DEGs in comparison of “LTP3_DC3000 vs WT_DC3000”. Nodes of KEGG pathway enriched with significantly upregulated DEGs were framed in red color, and downregulated DEGs in green color. Detailed information in **Table 1**.

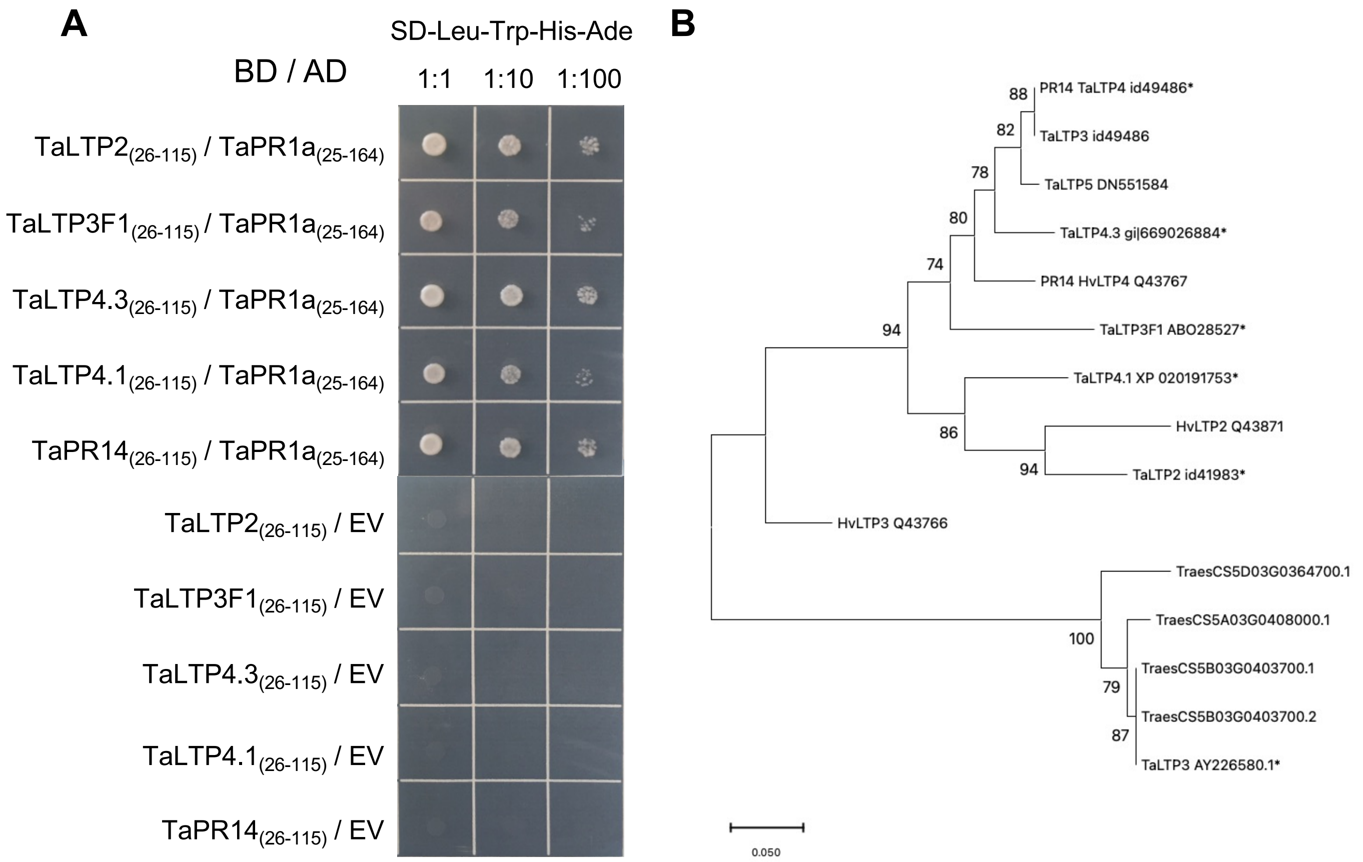

SUPPLEMENTARY FIGURE S3 | Interactions between TaPR1a and plant defense related LTP homologs. **(A)** Five wheat *LTP* homologous genes, including *TaLTP2*, *TaLTP3F1*, *TaLTP4.3*, *TaLTP4.1*, and *TaPR14* were cloned. The coding region of these genes excluding the corresponding predicted signal peptides were constructed into pGBKT7 (BD) vector. The recombinant constructs were co-transformed into yeast strain AH109 with TaPR1a$_{(25\text{-}164)}$-pGADT7 (AD) or EV-pGADT7 (control), respectively. The co-transformed yeast were assayed on SD-Leu-Trp-His-Ade selective medium. **(B)** Neighbor-joining tree for TaLTP3 and other plant defense related wheat LTP homologs. The polygenetic tree was generated by MEGA software. Values in the tree nodes indicate confidence values based on 1,000 bootstrap replications.

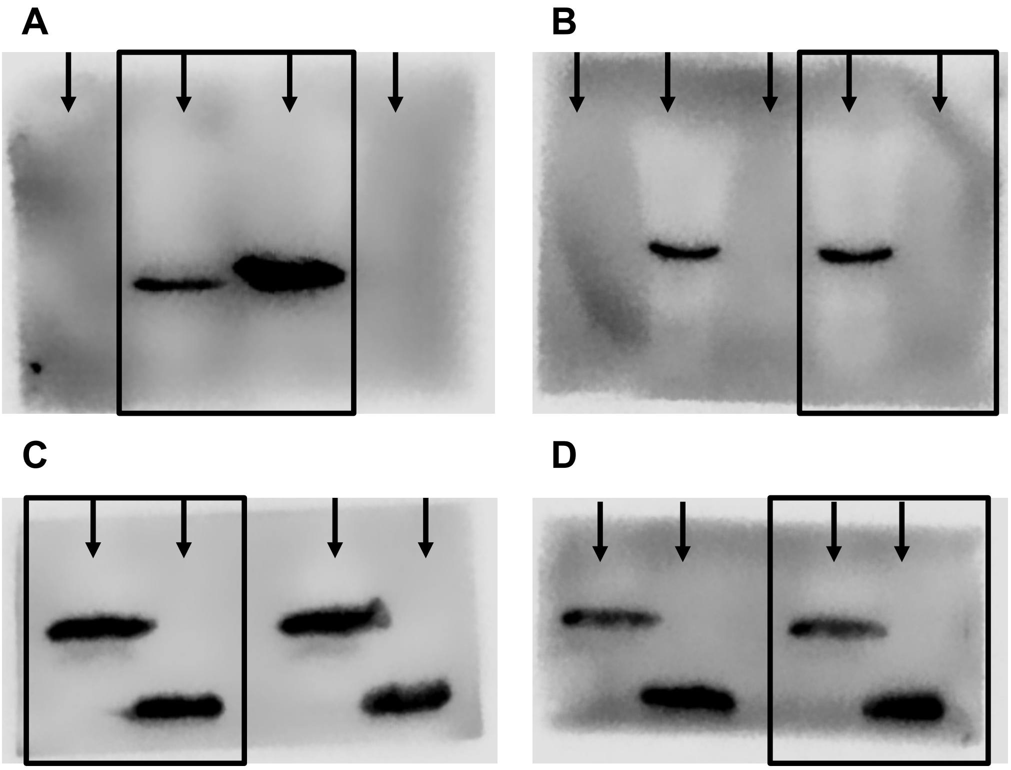

SUPPLEMENTARY FIGURE S4 | Original, uncropped blots for the Co-IP assay. (**A**) Immuno-blot of Input samples using α-cMYC antibody. Lanes 2 and 3 (from leaf to right) were cropped and annotated as the Left-UP panel of the **Figure 5D**. (**B**) Immuno-blot of α-GFP IP samples using α-cMYC antibody. Lanes 4 and 5 (from leaf to right) were cropped and annotated as the Right-UP panel of the **Figure 5D**. Other lanes were not relevant to this study. (**C**) Immuno-blot of Input samples using α-GFP antibody. Lanes 1 and 2 (from leaf to right) were cropped and annotated as the Left-Down panels of the **Figure 5D**. Other lanes were not relevant to this study. (**D**) Immuno-blot of α-GFP IP samples using α-GFP antibody. Lanes 3 and 4 (from leaf to right) were cropped and annotated as the Right-Down panels of the **Figure 5D**. Other lanes were not relevant to this study. Black arrows showed independent lanes and blots in frame were cropped.
