# Supplementary material for: Wheat Apoplast-Localized Lipid Transfer Protein TaLTP3 Enhances Defense Responses Against Puccinia triticina
Source: Front Plant Sci. 2021 Nov 25;12:771806. doi: 10.3389/fpls.2021.771806 (PMC8657149; doi:10.3389/fpls.2021.771806)
Supplement: Supplementary file 2 [file Data_Sheet_2.docx]

**SUPPLEMENTARY TABLE S1 |** Primers used in this study.

| **Primer usage** | **Primer name** | **Primer sequences 5^’^ to 3^’^** | **PCR product** | **Accession** | **Efficency** |
| --- | --- | --- | --- | --- | --- |
| Gene clone | TaLTP3-ORF-F | ATGGCTCGTCTCAACAGCAAGG | 369 bp | AY226580 | N.A. |
|  | TaLTP3-ORF-R | CTAGTTGACATTGTTGCAGTTGGTGGACA |  |  |  |
| Y2H | TaLTP3-nSP-F | caccGCGCTGTCGTGCGGGCA | 283 bp | AY226580 | N.A. |
|  | TaLTP3-nST-R | GTTGACATTGTTGCAGTTGGTGGACA |  |  |  |
|  | TaLTP2-nSP-F | caccGCCATCAGCTGCGGCCAG | 274 bp | AAK20395 | N.A. |
|  | TaLTP2-nST-R | GTGGATCTTAGAGCAGTCCACGGAT |  |  |  |
|  | TaLTP3F1-nSP-F | caccGCTGTATCGTGCGGTCAGGTGA | 274 bp | EF432573 | N.A. |
|  | TaLTP3F1-nST-R | GCGAATCTTAGAGCAGTCGACCG |  |  |  |
|  | TaLTP4.3-nSP-F | caccGCCATCTCCTGCGGTCAGGT | 274 bp | XP_020171778 | N.A. |
|  | TaLTP4.3-nST-R | GCGAATCTTAGAGCAGTCGACGG |  |  |  |
|  | TaLTP4.1-nSP-F | caccGCCATCTCCTGCGGTCAGGT | 274 bp | XP_020191753 | N.A. |
|  | TaLTP4.1-nST-R | GCGAATCTTAGAGCAGTCGACGG |  |  |  |
|  | TaPR14-nSP-F | caccGCGATATCCTGCGGTCAG | 274 bp | AFR54362 | N.A. |
|  | TaPR14-nST-R | ACGAATCTTAGAACAGTCCACAGA |  |  |  |
|  | TaPR1a-nSP-F | CACCgaattcCAGAACTCGCCTCAGGACTACC | 204 bp | FJ815169 | N.A. |
|  | TaPR1a-93aa-R | ggatccGTCCGCCGCCTTCCAGTCC |  |  |  |
|  | TaPR1a-128aa-R | ggatccCGCGCGCCACACCACCTG | 309 bp |  |  |
|  | TaPR1a-94aa-F | gaattcGACGCGGTGAAGCTGTGGGT | 210 bp |  |  |
| qRT-PCR | TaLTP3-qRT-F | GGCGGTGGTGCTGATGATGG | 145 bp | AY226580 | 99.4% |
|  | TaLTP3-qRT-R | TGATGGACTTGATGCTGGTGGC |  |  |  |
|  | TaPR1a-qRT-F | GCGGTGAAGCTGTGGGTGGA | 180 bp | FJ815169 | 94.4% |
|  | TaPR1a-qRT-R | GGGCTCGTAGTTGCAGGTGATGA |  |  |  |
|  | TaPR2-qRT-F | GTGGTGCAGCTCTACAGGTCCAA | 116 bp | CAA77085 | 93.0% |
|  | TaPR2-qRT-R | TTACCGATGTCGAGGATGAGGC |  |  |  |
|  | TaPR4b-qRT-F | TACGGGTGGACGGCGTTCTG | 177 bp | XP_020174891 | 92.0% |
|  | TaPR4b-qRT-R | GGTGTCGATCTTGGTGAAGACGGT |  |  |  |
|  | TaPAD4-qRT-F | GGAGGTTGTTGCGGTGATA | 150 bp | CJ934977 | 93.7% |
|  | TaPAD4-qRT-R | GCCAGTTTTGGGATGGTG |  |  |  |
|  | TaActin-qRT-F | ACCTTCAGTTGCCCAGCAAT | 91 bp | AB181991 | 102.6% |
|  | TaActin-qRT-R | CAGAGTCGAGCACAATACCAGTTG |  |  |  |
|  | TaPAL-qRT-F | GACCTCGTCCCGCTTTCCT | 252 bp | X99705 | 101.64% |
|  | TaPAL-qRT-R | GCCCGACAAGACCTCAGCA |  |  |  |
| BiFC and GFP | TaLTP3-ORF-F | caccATGGCTCGTCTCAACAGCAAGG | 370 bp | AY226580 | N.A. |
| Co-IP | TaPR1a-cMYC-R | ACTAGTttacaggtcctcctctgagatcagcttctgctcGTATGGTTTCTGTCCAATGACATTC | 453 bp | FJ815169 | N.A. |

**SUPPLEMENTARY TABLE S2 |** Raw data for evaluation of wheat resistance to *Pt* in *TaLTP3-OE* transgenic wheat lines.

| **Genotype** | **Rust uredinium / leaf region (%)** | **Biological replicates** | **Mean** | **SE** | **ANOVA** |
| --- | --- | --- | --- | --- | --- |
| WT | 7.86, 3.78, 3.68, 4.62, 2.47, 10.1, 9.18, 5.67, 8.95, 10.05, 3.18, 12.72, 4.89, 13.1, 17.15, 17.91, 3.74, 14.74, 5.91, 7.77, 7.02, 6.04, 7.29, 2.88, 13, 6.14, 6.08, 5.52, 3, 2.99 | 30 | 7.6 | 0.8 | N.A. |
| *TaLTP3-OE-E3-T1* | 0.6, 1.48, 1.39, 1.17, 0.58, 0.99, 1.8, 3.4, 0.63 | 9 | 1.3 | 0.3 | **<0.0001***** |
| *TaLTP3-OE-E22-T1* | 4.97, 6.89, 5.56, 6.95, 7.21, 6.8, 5.56, 1.72, 4.77, 0.28, 2.28, 1.9, 2.9 | 13 | 4.4 | 0.7 | **0.004**** |

**SUPPLEMENTARY TABLE S3 |** Sequencing information for the transcriptome.

| **Sample** | **raw_reads** | **clean_reads** | **clean_bases** | **error_rate** | **Q20** | **Q30** | **GC_pct** |
| --- | --- | --- | --- | --- | --- | --- | --- |
| WT_CK_1 | 91128880 | 88610906 | 13.29G | 0.02 | 98.15 | 95.12 | 58.77 |
| WT_CK_2 | 86809400 | 84158288 | 12.62G | 0.02 | 98.19 | 95.19 | 58.6 |
| WT_CK_3 | 84360440 | 81826754 | 12.27G | 0.02 | 98.26 | 95.36 | 58.43 |
| WT_DC_1 | 85545078 | 82602924 | 12.39G | 0.02 | 98.24 | 95.29 | 56.96 |
| WT_DC_2 | 89226750 | 86167538 | 12.93G | 0.03 | 97.89 | 94.22 | 57.26 |
| WT_DC_3 | 84458650 | 81910864 | 12.29G | 0.02 | 97.92 | 94.6 | 57.28 |
| LTP_CK_1 | 85931076 | 84474084 | 12.67G | 0.02 | 97.97 | 94.75 | 58.5 |
| LTP_CK_2 | 82989890 | 81097206 | 12.16G | 0.02 | 98.08 | 94.96 | 58.28 |
| LTP_CK_3 | 91688904 | 89010334 | 13.35G | 0.02 | 98.16 | 95.09 | 57.64 |
| LTP_DC_1 | 84567162 | 81989064 | 12.3G | 0.02 | 98.1 | 94.98 | 56.66 |
| LTP_DC_2 | 94161430 | 91283938 | 13.69G | 0.02 | 98.3 | 95.41 | 57.2 |
| LTP_DC_3 | 94536118 | 91685626 | 13.75G | 0.02 | 98.33 | 95.44 | 56.43 |

LTP: *TaLTP3-OE* transgenic wheat line, CK: mock-inoculation with water, DC: *P. syringae* DC3000 infection.

**SUPPLEMENTARY TABLE S4 |** Details of the transcriptome assembly.

| **sample** | **total_reads** | **total_map** | **unique_map** | **multi_map** | **read1_map** | **read2_map** | **positive_map** | **negative_map** | **splice_map** | **unsplice_map** | **proper_map** |
| --- | --- | --- | --- | --- | --- | --- | --- | --- | --- | --- | --- |
| WT_CK_1 | 88610906 | 84887241  (95.8%) | 80062165  (90.35%) | 4825076  (5.45%) | 39955775  (45.09%) | 40106390  (45.26%) | 40003371  (45.14%) | 40058794  (45.21%) | 27946129  (31.54%) | 52116036  (58.81%) | 76512858  (86.35%) |
| WT_CK_2 | 84158288 | 80644182  (95.82%) | 75862319  (90.14%) | 4781863  (5.68%) | 37841007  (44.96%) | 38021312  (45.18%) | 37912112  (45.05%) | 37950207  (45.09%) | 27379957  (32.53%) | 48482362  (57.61%) | 72492904  (86.14%) |
| WT_CK_3 | 81826754 | 78378170  (95.79%) | 73912998  (90.33%) | 4465172  (5.46%) | 36862374  (45.05%) | 37050624  (45.28%) | 36920198  (45.12%) | 36992800  (45.21%) | 25832533  (31.57%) | 48080465  (58.76%) | 70536936  (86.2%) |
| WT_DC_1 | 82602924 | 78752829  (95.34%) | 75148798  (90.98%) | 3604031  (4.36%) | 37493268  (45.39%) | 37655530  (45.59%) | 37538441  (45.44%) | 37610357  (45.53%) | 24531517  (29.7%) | 50617281  (61.28%) | 71876564  (87.01%) |
| WT_DC_2 | 86167538 | 82077178  (95.25%) | 78122127  (90.66%) | 3955051  (4.59%) | 39029126  (45.29%) | 39093001  (45.37%) | 39008899  (45.27%) | 39113228  (45.39%) | 25242884  (29.3%) | 52879243  (61.37%) | 74603906  (86.58%) |
| WT_DC_3 | 81910864 | 77981082  (95.2%) | 74284668  (90.69%) | 3696414  (4.51%) | 37111450  (45.31%) | 37173218  (45.38%) | 37118980  (45.32%) | 37165688  (45.37%) | 25653667  (31.32%) | 48631001  (59.37%) | 70808058  (86.45%) |
| LTP_CK_1 | 84474084 | 80630718  (95.45%) | 76306621  (90.33%) | 4324097  (5.12%) | 38086847  (45.09%) | 38219774  (45.24%) | 38126718  (45.13%) | 38179903  (45.2%) | 26956848  (31.91%) | 49349773  (58.42%) | 72497856  (85.82%) |
| LTP_CK_2 | 81097206 | 77537481  (95.61%) | 73293356  (90.38%) | 4244125  (5.23%) | 36588371  (45.12%) | 36704985  (45.26%) | 36623736  (45.16%) | 36669620  (45.22%) | 26071770  (32.15%) | 47221586  (58.23%) | 69927866  (86.23%) |
| LTP_CK_3 | 89010334 | 85248974  (95.77%) | 80690561  (90.65%) | 4558413  (5.12%) | 40290447  (45.26%) | 40400114  (45.39%) | 40293242  (45.27%) | 40397319  (45.38%) | 26802182  (30.11%) | 53888379  (60.54%) | 77243146  (86.78%) |
| LTP_DC_1 | 81989064 | 78220644  (95.4%) | 74460480  (90.82%) | 3760164  (4.59%) | 37168317  (45.33%) | 37292163  (45.48%) | 37208709  (45.38%) | 37251771  (45.44%) | 25350707  (30.92%) | 49109773  (59.9%) | 71299076  (86.96%) |
| LTP_DC_2 | 91283938 | 87119031  (95.44%) | 82933391  (90.85%) | 4185640  (4.59%) | 41500212  (45.46%) | 41433179  (45.39%) | 41448041  (45.41%) | 41485350  (45.45%) | 28728381  (31.47%) | 54205010  (59.38%) | 79369264  (86.95%) |
| LTP_DC_3 | 91685626 | 87236825  (95.15%) | 83015671  (90.54%) | 4221154  (4.6%) | 41547015  (45.31%) | 41468656  (45.23%) | 41463591  (45.22%) | 41552080  (45.32%) | 27950962  (30.49%) | 55064709  (60.06%) | 79527872  (86.74%) |

**SUPPLEMENTARY TABLE S5 |** Correlations of the overall gene expressions among biological replicates.

| **WT_CK_1** | **WT_CK_2** | **WT_CK_3** | **WT_DC_1** | **WT_DC_2** | **WT_DC_3** | **LTP_CK_1** | **LTP_CK_2** | **LTP_CK_3** | **LTP_DC_1** | **LTP_DC_2** | **LTP_DC_3** |
| --- | --- | --- | --- | --- | --- | --- | --- | --- | --- | --- | --- |
| **WT_CK_1** | 1 | 0.974 | 0.977 | 0.752 | 0.809 | 0.647 | 0.968 | 0.957 | 0.954 | 0.628 | 0.558 |
| **WT_CK_2** | 0.974 | 1 | 0.978 | 0.742 | 0.798 | 0.65 | 0.97 | 0.969 | 0.949 | 0.623 | 0.56 |
| **WT_CK_3** | 0.977 | 0.978 | 1 | 0.755 | 0.811 | 0.655 | 0.974 | 0.969 | 0.959 | 0.634 | 0.565 |
| **WT_DC_1** | 0.752 | 0.742 | 0.755 | 1 | 0.957 | 0.881 | 0.733 | 0.725 | 0.723 | 0.881 | 0.831 |
| **WT_DC_2** | 0.809 | 0.798 | 0.811 | 0.957 | 1 | 0.852 | 0.79 | 0.78 | 0.786 | 0.85 | 0.788 |
| **WT_DC_3** | 0.647 | 0.65 | 0.655 | 0.881 | 0.852 | 1 | 0.638 | 0.635 | 0.62 | 0.961 | 0.95 |
| **LTP_CK_1** | 0.968 | 0.97 | 0.974 | 0.733 | 0.79 | 0.638 | 1 | 0.978 | 0.972 | 0.628 | 0.556 |
| **LTP_CK_2** | 0.957 | 0.969 | 0.969 | 0.725 | 0.78 | 0.635 | 0.978 | 1 | 0.964 | 0.62 | 0.554 |
| **LTP_CK_3** | 0.954 | 0.949 | 0.959 | 0.723 | 0.786 | 0.62 | 0.972 | 0.964 | 1 | 0.619 | 0.538 |
| **LTP_DC_1** | 0.628 | 0.623 | 0.634 | 0.881 | 0.85 | 0.961 | 0.628 | 0.62 | 0.619 | 1 | 0.957 |
| **LTP_DC_2** | 0.558 | 0.56 | 0.565 | 0.831 | 0.788 | 0.95 | 0.556 | 0.554 | 0.538 | 0.957 | 1 |
| **LTP_DC_3** | 0.672 | 0.667 | 0.68 | 0.913 | 0.878 | 0.95 | 0.672 | 0.664 | 0.661 | 0.967 | 0.932 |

**SUPPLEMENTARY TABLE S6 |** DEGs of “WT_DC vs WT_CK” annotated in the KEGG pathway of “plant–pathogen interaction”.

| **KEGG Annotation** | **Gene ID** | **WT_CK**  **FPKM** | **WT_DC**  **FPKM** | **WT_DC vs WT_CK**  ***p*-adjust value** | **Gene Annotation** |
| --- | --- | --- | --- | --- | --- |
| CERK1 | TRIAE_CS42_6DL_TGACv1_527162_AA1699670 | 0.02 | 6.72 | 1.61×10^-18^ | Chitin elicitor receptor kinase |
|  | novel.5345 | 0.00 | 2.25 | 8.74×10^-10^ | LysM domain receptor-like kinase |
| elf 18 | novel.3014 | 0.51 | 1.38 | 5.18×10^-5^ | Elongation factor |
| CNGCs | TRIAE_CS42_2DL_TGACv1_160734_AA0553580 | 1.01 | 2.60 | 7.67×10^-8^ | Cyclic nucleotide-gated |
|  | TRIAE_CS42_7BL_TGACv1_579414_AA1907130 | 0.65 | 2.74 | 2.09×10^-13^ | Cyclic nucleotide-gated |
| FLS2 | TRIAE_CS42_2AL_TGACv1_094921_AA0304880 | 0.77 | 2.46 | 1.38×10^-3^ | LRR receptor |
| CDPK | TRIAE_CS42_2DL_TGACv1_158581_AA0522370 | 15.03 | 28.07 | 4.52×10^-3^ | Calcium-dependent protein kinase |
|  | TRIAE_CS42_4DS_TGACv1_361640_AA1171010 | 2.81 | 4.93 | 1.02×10^-2^ | Calcium-dependent protein kinase |
|  | TRIAE_CS42_6AS_TGACv1_485300_AA1542800 | 1.92 | 8.67 | 3.84×10^-16^ | Calcium-dependent protein kinase |
|  | novel.1228 | 7.71 | 16.39 | 4.12×10^-6^ | Calcium-dependent protein kinase |
|  | TRIAE_CS42_2AS_TGACv1_112475_AA0338720 | 3.18 | 5.95 | 2.69×10^-3^ | Calcium-dependent protein kinase |
|  | TRIAE_CS42_6DL_TGACv1_527968_AA1710340 | 0.36 | 1.56 | 8.44×10^-4^ | Calcium-dependent protein kinase |
|  | TRIAE_CS42_1BL_TGACv1_032657_AA0132500 | 4.07 | 11.98 | 7.40×10^-8^ | Calcium-dependent protein kinase |
| Rboh | TRIAE_CS42_5DL_TGACv1_433213_AA1405680 | 0.39 | 2.27 | 1.21×10^-5^ | Respiratory burst oxidase homolog protein |
|  | TRIAE_CS42_3DL_TGACv1_251733_AA0884390 | 0.06 | 0.29 | 1.32×10^-2^ | Respiratory burst oxidase homolog protein |
|  | TRIAE_CS42_4DL_TGACv1_343029_AA1127810 | 3.68 | 9.86 | 3.52×10^-4^ | Respiratory burst oxidase homolog protein |
| MPK | TRIAE_CS42_4BL_TGACv1_320270_AA1033300 | 5.67 | 13.89 | 1.12×10^-2^ | Mitogen-activated protein kinase |
| MKK | TRIAE_CS42_4BL_TGACv1_320624_AA1044950 | 1.80 | 4.07 | 5.84×10^-3^ | Mitogen-activated protein kinase kinase |
|  | TRIAE_CS42_7DS_TGACv1_623332_AA2052160 | 1.39 | 4.31 | 4.76×10^-5^ | Mitogen-activated protein kinase kinase |
| CAM | TRIAE_CS42_1BL_TGACv1_031230_AA0109990 | 0.24 | 1.61 | 4.23×10^-4^ | calcium-binding protein |
|  | TRIAE_CS42_3AL_TGACv1_194457_AA0633450 | 4.22 | 17.58 | 1.10×10^-4^ | calcium-binding protein |
|  | TRIAE_CS42_4DS_TGACv1_362249_AA1178220 | 0.01 | 13.16 | 1.45×10^-7^ | calcium-binding protein |
|  | TRIAE_CS42_2BS_TGACv1_146510_AA0467200 | 6.18 | 13.95 | 1.45×10^-6^ | calcium-binding protein |
|  | TRIAE_CS42_2AS_TGACv1_112492_AA0339230 | 89.73 | 196.76 | 8.74×10^-10^ | Calmodulin protein |
|  | TRIAE_CS42_3DS_TGACv1_271624_AA0904180 | 9.40 | 23.29 | 2.20×10^-6^ | Calmodulin protein |
|  | TRIAE_CS42_5AL_TGACv1_374264_AA1195470 | 0.03 | 11.28 | 1.11×10^-9^ | calcium-binding protein |
|  | TRIAE_CS42_1DL_TGACv1_063778_AA0230750 | 0.05 | 6.29 | 4.49×10^-7^ | calcium-binding protein |
|  | TRIAE_CS42_7BL_TGACv1_576890_AA1858760 | 0.00 | 3.37 | 1.23×10^-3^ | calcium-binding protein |
|  | TRIAE_CS42_7DS_TGACv1_623278_AA2051550 | 0.02 | 3.20 | 1.37×10^-4^ | calcium-binding protein |
|  | TRIAE_CS42_1BL_TGACv1_030243_AA0083380 | 0.00 | 8.53 | 6.30×10^-5^ | calcium-binding protein |
|  | TRIAE_CS42_4DL_TGACv1_342659_AA1119250 | 0.73 | 34.97 | 2.63×10^-26^ | calcium-binding protein |
| NHO1 | TRIAE_CS42_2DL_TGACv1_159098_AA0532320 | 4.20 | 10.78 | 1.10×10^-7^ | glycerol phosphotransferase |
| PR1 | novel.4381 | 0.00 | 10.48 | 1.20×10^-3^ | Pathogenesis-related protein |
|  | TRIAE_CS42_5DL_TGACv1_433848_AA1423710 | 0.00 | 0.31 | 4.62×10^-2^ | Pathogenesis-related protein |
|  | TRIAE_CS42_5BL_TGACv1_405157_AA1321310 | 0.56 | 18.54 | 2.60×10^-7^ | Pathogenesis-related protein |
| WRKY | TRIAE_CS42_3B_TGACv1_221765_AA0749500 | 1.52 | 16.07 | 1.84×10^-23^ | WRKY transcription factor |
|  | TRIAE_CS42_1AS_TGACv1_019570_AA0068550 | 1.56 | 15.84 | 6.56×10^-12^ | WRKY transcription factor |
|  | TRIAE_CS42_1BL_TGACv1_031863_AA0121910 | 0.24 | 7.36 | 8.85×10^-22^ | WRKY transcription factor |
| Pti1 | TRIAE_CS42_2DL_TGACv1_158379_AA0516920 | 3.39 | 7.44 | 1.39×10^-4^ | PTI-like tyrosine-protein kinase |
|  | TRIAE_CS42_1AS_TGACv1_019980_AA0073520 | 3.52 | 10.79 | 6.11×10^-12^ | Probable receptor-like protein kinase |
|  | TRIAE_CS42_5BL_TGACv1_406045_AA1339760 | 3.61 | 5.85 | 6.42×10^-4^ | PTI-like tyrosine-protein kinase |
| RIN4 | TRIAE_CS42_2DL_TGACv1_159392_AA0537640  TRIAE_CS42_5BL_TGACv1_407607_AA1358320  TRIAE_CS42_U_TGACv1_642291_AA2115230 | 6.20 | 9.15 | 1.46×10^-2^ | Avr Cleavage site |
|  |  | 3.44 | 11.50 | 6.70×10^-16^ | RPM1-interacting protein |
|  |  | 2.63 | 13.88 | 8.65×10^-18^ | RPM1-interacting protein |
| RPM1 | TRIAE_CS42_4AL_TGACv1_289889_AA0978430 | 1.81 | 3.24 | 6.66×10^-4^ | Disease resistance protein |
|  | TRIAE_CS42_2BS_TGACv1_147972_AA0489490 | 0.88 | 3.20 | 3.79×10^-10^ | Disease resistance protein |
|  | TRIAE_CS42_5AL_TGACv1_379686_AA1256710 | 0.02 | 0.22 | 4.45×10^-3^ | Disease resistance protein |
| RPS2 | TRIAE_CS42_3AL_TGACv1_196436_AA0660360 | 0.11 | 0.50 | 2.46×10^-5^ | Disease resistance protein |
|  | TRIAE_CS42_2DL_TGACv1_160613_AA0552330 | 0.06 | 2.67 | 4.93×10^-6^ | Disease resistance protein |
| PBS1 | TRIAE_CS42_2DL_TGACv1_162013_AA0561490 | 8.45 | 15.78 | 1.43×10^-4^ | Serine/threonine protein kinase |
| SGT1 | TRIAE_CS42_3AL_TGACv1_195352_AA0648390 | 10.23 | 22.95 | 3.67×10^-10^ | Protein SGT1 homolog |
| RAR1 | TRIAE_CS42_2BL_TGACv1_131024_AA0421420 | 2.52 | 6.48 | 7.20×10^-5^ | Cysteine/histidine domain protein |
| HSP90 | novel.1308 | 1.06 | 1.99 | 4.89×10^-3^ | Heat shock cognate protein |
|  | TRIAE_CS42_7AL_TGACv1_556087_AA1755000 | 4.72 | 39.58 | 1.93×10^-39^ | Glu regulated protein homolog |
|  | TRIAE_CS42_5BL_TGACv1_408217_AA1362310 | 31.88 | 52.46 | 1.31×10^-3^ | Heat shock cognate protein |
|  | TRIAE_CS42_5DS_TGACv1_456483_AA1471770 | 1.53 | 3.00 | 6.60×10^-4^ | Heat shock cognate protein |
|  | TRIAE_CS42_7DS_TGACv1_622763_AA2044980 | 7.23 | 43.30 | 8.59×10^-45^ | Heat shock cognate protein |
|  | TRIAE_CS42_7BS_TGACv1_593752_AA1954160 | 4.59 | 39.61 | 5.94×10^-61^ | Heat shock cognate protein |
| KCS1/FDH | TRIAE_CS42_1DL_TGACv1_062975_AA0222420 | 2.15 | 3.25 | 4.34×10^-2^ | long-chain fatty acid condensing enzyme |
|  | novel.6167 | 0.07 | 1.45 | 1.79×10^-6^ | long-chain fatty acid condensing enzyme |

CK: mock-inoculation with water, DC: *P. syringae* DC3000 infection.

**SUPPLEMENTARY TABLE S7 |** A summary of H_2_O_2_ accumulations induced by *P. syringae* DC3000 in wheat transgenic lines.

| **Genotype** | **2 hpi** | | | **4 hpi** | | | **8 hpi** | | |
| --- | --- | --- | --- | --- | --- | --- | --- | --- | --- |
|  | **Percentage of DAB stained areas**  **Mean ± SE** | **Number of biological replicates** | ***P* value** | **Percentage of DAB stained areas**  **Mean ± SE** | **Number of biological replicates** | ***P* value** | **Percentage of DAB stained areas**  **Mean ± SE** | **Number of biological replicates** | ***P* value** |
| WT | 19.7 ± 2.2 | 10 | N.A. | 27.7 ± 2.4 | 10 | N.A. | 25.2 ± 2.1 | 10 | N.A. |
| *TaPR1a-OE*-E4-T1 | 23.7 ± 3.1 | 10 | 0.3812 | 40.7 ± 2.3 * | 10 | 0.0409 | 45.8 ± 6.5 ** | 10 | 0.0003 |
| *TaLTP3-OE*-E1-T1 | 12.9 ± 3.8 | 7 | 0.0946 | 38.7 ± 5.1 ** | 5 | 0.0070 | 53.0 ± 3.8 ** | 7 | 0.0043 |
| WT | 12.8 ± 1.8 | 8 | N.A. | 29.7 ± 3.2 | 8 | N.A. | 22.8 ± 4.7 | 8 | N.A. |
| *TaPR1a-OE*-E1-T1 | 19.6 ± 3.1 | 8 | 0.085 | 42.6 ± 3.3 * | 8 | 0.0146 | 23.1 ± 3.4 | 6 | 0.9561 |
| *TaLTP3-OE*-E3-T1 | 30.9 ± 4.0 ** | 8 | 0.0021 | 29.8 ± 5.1 | 8 | 0.9764 | 23.4 ± 2.4 | 8 | 0.9097 |

**SUPPLEMENTARY TABLE S8 |** A summary of O_2_^-^ accumulations induced by *P. syringae* DC3000 in wheat transgenic lines.

| **Genotype** | **2 hpi** | | | **4 hpi** | | | **8 hpi** | | |
| --- | --- | --- | --- | --- | --- | --- | --- | --- | --- |
|  | **Percentage of NBT stained areas**  **Mean ± SE** | **Number of biological replicates** | ***P* value** | **Percentage of NBT stained areas**  **Mean ± SE** | **Number of biological replicates** | ***P* value** | **Percentage of NBT stained areas**  **Mean ± SE** | **Number of biological replicates** | ***P* value** |
| WT | 40.6 ± 5.1 | 7 | N.A. | 18.6 ± 4.5 | 7 | N.A. | 17.2 ± 2.6 | 7 | N.A. |
| *TaPR1a-OE-E1-T1* | 75.0 ± 4.6** | 8 | 0.0003 | 79.0 ± 6.1** | 8 | <0.0001 | 10.4 ± 2.0 | 8 | 0.0644 |
| *TaLTP3-OE-E3-T1* | 34.6 ± 3.5 | 8 | 0.3602 | 41.0 ± 7.1* | 8 | 0.0210 | 15.0 ± 5.2 | 8 | 0.7203 |
| WT | 73.2 ± 3.2 | 7 | N.A. | 27.2 ± 4.1 | 7 | N.A. | 10.3 ± 1.7 | 7 | N.A. |
| *TaPR1a-OE-E4-T1* | 94.5 ± 9.2* | 8 | 0.0391 | 42.7 ± 5.3 | 8 | 0.0929 | 10.4 ± 2.4 | 8 | 0.9844 |
| *TaLTP3-OE-E1-T1* | 81.7 ± 15.5 | 8 | 0.3887 | 42.0 ± 9.4 | 8 | 0.2235 | 11.8 ± 2.1 | 8 | 0.5765 |
